# Acute Liver Failure

**DOI:** 10.1155/1999/74658

**Published:** 1999-07

**Authors:** William M. Lee, Roger Williams


*HPB Surgery*, 1999, Vol. 11, p. 283
Reprints available directly from the publisher
Photocopying permitted by license only

© 1999 OPA (Overseas Publishers Association) N.V.
Published by license under
the Harwood Academic Publishers imprint,
part of The Gordon and Breach Publishing Group.
Printed in Malaysia.

### *Book Review*

**ACUTE LIVER FAILURE, Edited by William M. Lee and Roger Williams, ISBN 0 521 55381 4 (hardback) Price £ 70,00 (US $ 100, 00).**

Acute liver failure is a highly complex syndrome which afflicts many patients. It is a distinct clinical syndrome which crosses medical disciplines. It is therefore logical that the syndrome should be presented in a multi authorship book like this under the editorship of William M. Lee and Roger Williams. The authors have in this volume of 23 independent chapters given an excellent overview of the present knowledge of this syndrome. The first parts includes chapters on the clinical syndrome and aetiology and they are followed by mechanisms of the disease and multi system involvement. This part is up to date with a clarification of the role of cytokines and knowledge on hepatocyte replication and liver regeneration. This section carries its subject in an excellent overview up to present day knowledge. Thereafter follows four chapters on intensive care management with very clear guidelines for the work-up of diagnosis and verification of prognostic factors and the treatment of circulatory problems as well as pulmonary oedema and intracranial hypertension. In this chapter all the different pathogenetic mechanisms are clarified although I miss some information on the false neurotransmitters and derangement of the normal neurotransmitter profile. Infectious complications in liver failure are very common and therefore management of infection in acute liver failure deserves a chapter of itself. In this chapter the authors clarify the role of gut bacteria in liver failure and different ways of reducing septic insults. They rightly point out that other measures must be taken than massive antibiotic therapy and gut decontamination. Such measures are the use of growth factors and other factors influencing the granulocyte function. Acute liver transplantation in liver failure has been used with great success. This problem is dealt with in the coming sections with European and American experience.

The most fascinating development right now is on artificial and bioartificial liver devices, and the final chapters are devoted to this subject. By a clever use of hepatocytes a bioreactor system can be developed and put into action together with charcoal filtration and in this way patients can be treated for several days. This can serve as a bridge to liver transplantation.

This book will certainly be important for the management of patients with acute liver failure, but it will also defend its place in the library since it deals so much also with pathophysiology and aetiology.

BENGT JEPPSSON
Professor of Surgery

